# Adding functional properties to beer with jasmine tea extract

**DOI:** 10.3389/fnut.2023.1109109

**Published:** 2023-03-03

**Authors:** De-Quan Chen, Chun Zou, Yi-Bin Huang, Xuan Zhu, Patrizia Contursi, Jun-Feng Yin, Yong-Quan Xu

**Affiliations:** ^1^Tea Research Institute Chinese Academy of Agricultural Sciences, National Engineering Research Center for Tea Processing, Key Laboratory of Tea Biology and Resources Utilization, Hangzhou, China; ^2^Graduate School of Chinese Academy of Agricultural Sciences, Beijing, China; ^3^College of Tea Science, Guizhou University, Guiyang, China; ^4^School of Food and Bioengineering, Zhejiang Gongshang University, Hangzhou, China; ^5^Department of Biology, University of Naples Federico II, Naples, Italy

**Keywords:** jasmine tea extract, hop, volatile components, brewing process, tea beer

## Abstract

Hops provide the characteristic bitter taste and attractive aroma to beer; in this study, hops were replaced by jasmine tea extract (JTE) during late-hopping. The addition of JTE improved the beer foam stability 1.52-fold, and increased the polyphenol and organic acid contents. Linalool was the most important aroma compound in hopped (HOPB) and jasmine tea beer (JTB), but other flavor components were markedly different, including dimeric catechins, flavone/flavonol glycosides, and bitter acids and derivatives. Sensory evaluation indicated that addition of JTE increased the floral and fresh-scent aromas, reduced bitterness and improved the organoleptic quality of the beer. The antioxidant capacity of JTB was much higher than that of HOPB. The inhibition of amylase activity by JTB was 30.5% higher than that of HOPB. Functional properties to beer were added by substituting jasmine tea extract for hops during late hopping.

## 1. Introduction

Beer is one of the most widely consumed alcoholic beverages worldwide; water, malt, hops, and yeast are the four main ingredients used in the production of beer (1). Beer production comprises several stages, namely, mashing, boiling, fermentation, maturation and solid liquid separations (wort separation, wort clarification, and rough beer clarification) (2). In recent years, consumer preference has increasingly emphasized the flavor and nutritional qualities of beer, which has increased interest and demand for craft beers (3). The main differences between craft and mass-produced beer are a more concentrated raw wort, the addition of a greater variety and quantity of hops, or the addition of other auxiliary flavor/nutritional ingredients, such as fruits, tea and natural plant extracts. The flavor of beer is influenced by the ingredients, particularly by auxiliary ingredients, the yeast, the brewing process and the fermentation conditions (4–7).

Hops (*Humulus lupulus* L.) are an essential ingredient in beer manufacturing, providing the characteristic bitter taste and attractive aroma of the final beverage (8). The main volatile component of hops is its essential oil, 80% of which is composed of myrcene, α-humulene, and β-caryophyllene (9), however, these volatiles contribute little to the beer aroma, because of their low water solubility and their tendency to oxidize and evaporate during heating and fermentation (10). Hops contain numerous bicyclic and tricyclic minor terpene hydrocarbons, the most important of which are the monoterpene alcohols, linalool and geraniol, as well as their isomers nerol and α-terpineol (11, 12), which impart floral, geranium-like, fresh, and citrus notes to beer (13), contribute to inhibiting beer spoilage bacteria, and improving taste and foam stability (11). There are three main ways of adding hops (i.e., kettle, late, or dry hopping), late hopping and dry hopping have become key tools for brewers to impart beer with an intense hoppy aroma (12, 13). Late hopped beers have more pronounced hoppy and herbal aromas than early hopped ones, and late hopping increases the content of linalool and geraniol (14–16); delaying the addition of hops increases the geraniol content of the finished beer while avoiding conversion of geraniol to β-citronellol by the yeast (17).

Craft beer brewers often enrich the flavor of their beers by adding accessory ingredients, such as fruit, tea, and stevia to enhance consumer appeal. Addition of persimmon juice enhanced the antioxidant properties and consumer preference for the beer (18). The addition of white grape pomace increased the concentration of many volatile compounds in the beer, such as ethyl decanoate and ethyl dodecanoate, as well as increasing the phenolic content and antioxidant capacity (19). Addition of three types of tea (green, black, and oolong tea) increased the concentrations of tea volatile components, such as methyl salicylate, indole, and geraniol and its derivatives (20). Olive leaves have also been used in place of hops, to provide bitterness (21). Tea therefore has the potential to replace hops and provide the beer with a desirable, but unusual flavor.

The main objectives of this study were to enrich the flavor of beer by replacing hops with Jasmine tea extract (JTE) during late hopping and to investigate the effects of this on the quality of beer. This process modification has the potential to develop a novel application for tea and compensate for the scarcity of aromatic hops in China.

## 2. Materials and methods

### 2.1. Chemicals and standards

3-Nonanone (98%, Heowns, Tianjin, China), organic acid standards (oxalic acid, tartaric acid, malic acid, lactic acid, acetic acid, citric acid and succinic acid) were from Shanghai yuanye Bio-Technology Co., Ltd. Catechin standards (C, catechin; EC, epicatechin; GC, gallocatechin; CG, catechin gallate; EGC, epigallocatechin; CAF, caffeine; ECG, epicatechin gallate; EGCG, epigallocatechin gallate.) were from Sigma-Aldrich (Shanghai, China). *Saccharomyces cerevisiae* (lager yeast, s-33) was from Fermentis (Marquette-lez-Lille, France). 2,2-Diphenyl-1-picrylhydrazyl (DPPH), 2,2-azino-bis (3-ethylbenthiazoline-6-sulfonic acid (ABTS), and tripyridyl triazine (TPTZ) were from Sigma-Aldrich (Shanghai, China). All other chemicals and solvents used were of analytical grade, from local suppliers.

### 2.2. Brewing process

HOPB and JTB were produced in a 300 L pilot-scale brewing plant (Zhejiang Gongshang University). Pilsner-type (75 kg) and wheat (25 kg) malt was crushed using a two-roll mill and then transferred to a stainless-steel mashing vessel and mixed with water (45°C, 300 L). The process was initiated with the mash-in at 45°C (20 min), and the temperature was then gradually increased and maintained at 50°C (30 min), 65°C (40 min), 72°C (20 min) for maltose saccharification and 78°C (10 min) for enzyme inactivation, respectively. After complete mash conversion, the sweet wort was separated from the spent grains by lautering, then transferred to the kettle for boiling. The wort was boiled for 110 min with the addition of hops pellet (100 g, alfa-acid proportion: 13%) after 30 min and jasmine tea extract (360 g) after 100 min. The coarse break was separated through settling and the wort was cooled to about 40°C and transferred to the fermentation tank where the brewing yeast (150 g/L, activated for 30 min in sterile water) was added. After 7 days of primary fermentation at 20°C, the yeast slurry is drained from the bottom of the fermenter, followed by a temperature adjustment to 4°C and a closed venting valve for post-fermentation and maturation for 60 days, promptly sampling, storage (−80°C) and analysis. The control samples were brewed in the same way except for the addition of different ingredients at the late hopping stage. For the control sample, 150 g (alfa-acid proportion: 13%) of hops were added after 100 min of boiling.

### 2.3. Physicochemical analyses

Beer analysis was performed after maturation, following procedures in Chinese standard GB/T4928-2008. Color was determined by the spectrophotometric method (5.6.2) (Unico-2000, Unico, Shanghai, China); foam stability with a foam measuring cup (7.2); total acid content by titration with 0.1 mol/L NaOH; ethanol concentration was determined by quantitative distillation according to Dietz et al. (12). The alcohol concentration was determined with an SBA-40E biosensor (Shandong Biosensor Institute, China). Turbidity was determined using a HACH-TL2300 Turbidity Meter (HACH, Shanghai, China, detection limit = 0.001 NTU). A pH meter (FivrGo-2, Mettler Toledo, Shanghai, China) was used for pH measurements.

### 2.4. Characterization of tea catechins by HPLC

HPLC was used to analyze some targeted components, such as GA, GC, EGC, C, EC, EGCG, GCG, ECG, and caffeine, as described previously (22, 23). The HPLC (Agilent Technologies, Santa Clara, CA) was equipped with an Infinity binary pump, an autosampler, a column thermostat (set at 30°C), a diode array detector and an Agilent Zorbax SB-Aq C18 column (250 × 4.6 mm i.d., 5 μm). The mobile phases were 0.2% v/v aqueous formic acid (A) and methanol (B). The initial solvent was 5% B, which was ramped linearly to 20% B at 5 min, 25% B at 18 min, 42% B at 25 min, held for 7 min, then increased to 100% B at 40 min. The total run time was 40 min, the flow rate 1.0 ml/min, the injection volume 5 μl and the detection wavelength 278 nm.

### 2.5. Headspace-solid phase microextraction (HS-SPME) and GC-MS analysis of beer

The analysis was performed as described previously (24). Bottles of beer were maintained at 4°C to minimize loss of volatiles. Beer sample (4 ml), water (4 ml), internal standard (3-Nonanone, 20 μl, 9.8 mg/L) and NaCl (1 g) were added to 20 ml SPME headspace vials and sealed with a polytetrafluoroethylene (PTFE)-silicon septum. The septum covering the vial headspace was pierced with the needle containing the SPME fiber and retracted, and the fiber was exposed to the headspace for 30 min at 50°C, then inserted directly into the GC-MS injection port. The carrier gas was helium at a flow rate of 1 ml/min. Samples were analyzed on a DB-5MS UI column (30 m by 0.250-mm inside diameter by 0.25-μm film thickness, Agilent). The oven temperature was programmed as follows: initial temperature 35°C, held for 5 min, increased at 4°C/min to 130°C, held for 3 min, then at 5°C/min to 230°C, held for 5 min. Electron impact (EI) ionization was used at 70 eV, scanning from *m*/*z* 10 to 250. Background subtraction was performed on the raw GC-MS data using data processing software. The National Institute of Standards and Technology (NIST 14) database was used for qualitative analysis of the MS peaks corresponding to the chromatographic peak signals at different retention times (>70%). The peaks were quantified by comparison with the internal standard (3-Nonanone) to calculate the relative content of each substance and the data were imported into Simca-P software (Version 14.1, MKS Umetrics AB, Umeå, Sweden). The outputs were subjected to Orthogonal partial least squares-discriminant analysis (OPLS-DA). The variable importance in projection (VIP) value was used to evaluate data generated by OPLS-DA; only data with VIP values >1 were selected for further analysis.

### 2.6. Non-targeted metabolomics analysis

Non-targeted metabolomics analysis was carried out using UPLC-HRMS (Q-Exactive system, Thermo Fisher Scientific, Rockford, IL), as described previously (25) with some modifications. The column was an Acquity UPLC BEH C18 (100 nm × 2.1 mm; 1.7 μm, Waters, Manchester, UK). The mobile phases were aqueous, 0.1% v/v formic acid (A) and acetonitrile (B), and the linear elution gradient program was 0–1.0 min, 5% B; 2.0 min, 10% B; 6.0 min, 35% B; 8.5–9.5 min, 100% B; and 10.0–12.0 min, 5% B. The total analysis time was 12 min and the flow rate was 0.3 ml/min. The column and autosampler were set at 40 and 10°C, respectively.

Mass spectrometric analysis was performed with the Q-Exactive Orbitrap mass analyzer in negative ionization mode at a spray voltage of 3.0 kV. The capillary temperature and auxiliary gas heater temperature were both 300°C. The flow rates of sheath gas and auxiliary gas were set to 45 and 10 arbitrary units, respectively. The full scan MS/data-dependent MS/MS (ddMS2) mode was used, in which the resolution was 70,000 and 35,000 for full MS and ddMS2, respectively. The mass scan range was from *m*/*z* 66.7 to 1000.

All the samples were filtered through a 0.45 μm Millipore filter. The raw data acquired were processed on Compound Discoverer software (Version 3.0, Thermo Fisher) to obtain all the ion fragment information through peak picking and alignment. This information was used for partial least-squares discriminant analysis (PLS-DA) to screen for differential metabolites with VIP >1.2 and *p* < 0.05, which was performed on Simca-P v14.1. Thereafter, the Human Metabolome Database (http://www.hmdb.ca/), our laboratory's standards library and previous metabolomics studies (26, 27) were used for identification of the differential metabolites.

### 2.7. Total polyphenol content

The total phenolic content of beer was determined spectrophotometrically with Folin–Ciocalteu reagent (28). A calibration curve was plotted using gallic acid as standard. The beer samples were diluted with deionized water to adjust the concentration of phenolic compounds to the linear calibration range of gallic acid. Results were expressed as mg of gallic acid equivalent (GAE) per liter.

### 2.8. Antioxidant capacity

#### 2.8.1. DPPH radical scavenging capacity

The DPPH radical scavenging capacity was determined as described previously (29, 30). The samples were appropriately diluted with ethanol, then sample (2 ml) was mixed with DPPH (2 ml, 0.2 mmol/L), then left to stand for 30 min at room temperature in the dark. The absorbance was measured with a spectrophotometer at 517 nm using quartz cuvettes (A_S_). Ethanol was used as a blank control (A_0_). DPPH radical scavenging capacity was calculated as:


$$\begin{array}{l} \text{DPPH\%}=\frac{{\text{A}}_{\text{s}}-{\text{A}}_{0}}{{\text{A}}_{\text{s}}}\times 100 \end{array}$$

where A_0_ is the absorbance of blank and A_S_ is the absorbance of the sample.

#### 2.8.2. ABTS radical scavenging capacity

The ABTS radical scavenging capacity (ABTS%) was determined as described previously (31), with some modifications. The ABTS stock solution was made by mixing equal amounts of 14 mmol/L ABTS solution and 4.9 mmol/L potassium persulfate solution and stored for 14–16 h in the dark at room temperature to generate ABTS radicals, then diluted with methanol to achieve an absorbance of 0.75 ± 0.05 at 734 nm. Diluted ABTS radical solution (3.9 ml) was mixed with 0.1 ml of sample solution and left at room temperature in darkness for 6 min before reading its absorbance at 734 nm. Deionized water was used as the blank (A_0_). ABTS% was calculated using the equation:


$$\begin{array}{l} \text{ABTS\%}=\frac{{\text{A}}_{\text{S}}-{\text{A}}_{0}}{{\text{A}}_{\text{S}}}\times 100 \end{array}$$

where A_0_ is the absorbance of the blank and A_S_ is the absorbance of the sample.

#### 2.8.3. Ferric reducing antioxidant power

Ferric reducing antioxidant power (FRAP) assays were performed as described previously (32). The FRAP solution was prepared by mixing acetate buffer (300 mmol/L, pH 3.6), tripyridyltriazine (TPTZ 10 mmol/L) and FeCl_3_ solution (20 mmol/L) in a ratio of 10:1:1 (v/v/v). An aliquot (100 μl) of sample solution was mixed with FRAP solution (3 ml) and incubated at 37°C for 10 min, then the absorbance at 593 nm was measured. Quantitation was achieved with reference to a standard curve of FeSO_4_ (0.2–0.8 mmol/L) and results are expressed as millimoles (mmol) of Trolox per liter of beer.

### 2.9. α-Amylase inhibition assay

α-Amylase inhibition was determined as described previously (33), with minor modifications. Test samples (200 μl) were added to sodium phosphate buffer (300 μl, 0.02 M pH 6.9) containing 30 unit/ml porcine pancreatic α-amylase and preincubated at 37°C for 10 min. After preincubation, starch solution (300 μl, 0.5%) was added, then the reaction mixtures incubated at 37°C for 15 min. The reaction was stopped by adding 3,5-dinitrosalicylic acid reagent (600 μl), heating in a boiling water bath for 10 min, then cooling. After adding 900 μl of distilled water, the absorbance was measured at 540 nm. The α-amylase inhibitory activity was calculated as follows:


$$\begin{array}{l} \text{Inhibitory activity }(\%)\;=\;[1-\;({\text{OD}}_{\text{test sample}}\;-\;{\text{OD}}_{\text{blank}})\\ \text{ / }{\text{OD}}_{\text{control}}]\;\times \text{100} \end{array}$$

### 2.10. α-Glucosidase inhibition assay

The α-glucosidase inhibition was determined as described previously (34) with minor modifications. α-Glucosidase (100 μl, 70 U/ml, in 0.5 ml sodium acetate buffer, pH 5.0) was premixed with beer sample (0.5 ml) and incubated at 37°C for 15 min. *p*-Nitrophenyl-α-D-glucopyranoside (0.5 ml, 2.5 mmol/L) was added, then the mixture was incubated at 37°C for 15 min and stopped by adding sodium carbonate solution (1 ml 0.2 mol/L). The α-glucosidase activity was determined by measuring the release of *p*-nitrophenol at 405 nm. α-Glucosidase inhibition was calculated as follows:


$$\begin{array}{l} \text{Inhibitory activity }(\%)\;=\;[1-\;({\text{OD}}_{\text{test sample}}\;-\;{\text{OD}}_{\text{blank}})\\ \text{ / }{\text{OD}}_{\text{control}}]\;\times \text{100} \end{array}$$

### 2.11. Sensory analysis

The beers were evaluated by 29 untrained tasters from the Tea Research Institute, Chinese Academy of Agricultural Sciences, aged between 20 and 59 years. For each taster, a 40 ml sample of the beer was served in a disposable, clear, acrylic glass. The tasters evaluated the aroma, taste, foam, appearance and overall score using a nine point hedonic scale form, where 1 = dislike strongly; 5 = neither like, nor dislike; and 9 = like strongly (35). A second four level scale (not sensed, faintly sensed, mildly sensed, and strongly sensed) was used to grade bitter and astringent tastes, and malty aroma, fruital aroma, floral aroma, and fresh-scent aroma. Training of tasters in grade evaluation prior to the experiment. All the participants (healthy and non-smokers from TRICAAS) were conducted considering the principle outlined in the Declaration of Helsinki, and informed written consent was obtained.

### 2.12. Statistical data analysis

The data are presented as the mean ± standard error of the mean. All experiments were carried out in triplicate. The results were analyzed with SPSS 18.0, using one-way analysis of variance to determine differences between sample groups, with *p* < 0.05 considered statistically significant.

## 3. Results and discussion

### 3.1. Physicochemical properties of beers

The effects of JTE on the physicochemical properties of the beers were compared (Table 1). JTE addition did not affect (*p* > 0.05) the color, turbidity and diacetyl content, however, it affected (*p* < 0.05) the contents of alcohol, total acids, total polyphenols, total catechins, and the foam-stability. The foam stability, alcohol concentration, total polyphenol concentration, and total catechin concentration of JTB were 528.0 ± 22.7 s, 6.6 ± 0.14% (v/v), 921.95 ± 1.6 mg/L, and 302.4 ± 1.3 mg/L, respectively, 1.52-, 1.04-, 1.6-, and 34.72-fold those of HOPB, respectively.

**Table 1:** Basic physico-chemical parameters of the beer.

**Items**	**HOPB**	**JTB**
Color (EBC)	15.73 ± 1.17	15.12 ± 2.68
Turbidity (EBC)	2.89 ± 0.01	2.93 ± 0.03
Foam-stability (s)	347.67 ± 27.54^b^	528.00 ± 22.65^a^
Alcoholic content (%, v/v)	6.38 ± 0.21	6.63 ± 0.14
Diacetyl content (mg/L)	0.08 ± 0.01	0.06 ± 0.03
Total acids content (ml/100 ml)	3.35 ± 0.08^b^	3.75 ± 0.30^a^
Total polyphenols content (mg/L)	576.76 ± 4.86^b^	921.94 ± 1.55^a^
Total catechins content (mg/L)	8.71 ± 0.03^b^	302.39 ± 1.29^a^

Data are means (±SD) of three replicates.

EBC, European Brewery Convention units.

^a, b^Different letters in the same row indicate significant differences between mean values (p < 0.05).

The catechin concentration of JTB was markedly higher than that of HOPB (Table 2), because of the high catechin concentration of JTE (17), and since catechins are the major JTE polyphenols, the polyphenol content of JTB was also significantly higher than that of HOPB. The foam stability of JTB was significantly higher, which may be related to its greater polyphenol content (18) and the foam-stabilizing effect of some polyphenols (36). Studies have shown that the type and content of polyphenols and catechins in beer are important factors affecting the antioxidant capacity and flavor stability of beer (37). In this study, it was found that beers with added tea extracts had stronger antioxidant capacity. Taken together, these results suggest that there is an association between JTE addition and beer quality.

**Table 2:** Content of catechin-like compounds in beer.

	**JTB**	**HOPB**
GA	25.24 ± 0.02^a^	N
GC	2.08 ± 0.04^a^	N
EGC	49.79 ± 3.10^a^	N
C	49.77 ± 3.19^a^	1.90 ± 0.03^b^
EGCG	62.02 ± 0.33^a^	N
EC	54.29 ± 0.43^a^	6.81 ± 0.05^b^
GCG	12.48 ± 2.07^a^	N
ECG	9.13 ± 1.39^a^	N
CG	37.58 ± 1.07^a^	N

Data are means (±SD) of three replicates.

N, not detected.

^a, b^Different letters in the same row indicate significant differences between mean values (p < 0.05).

### 3.2. Organic acid content and composition

**Figure 1:**
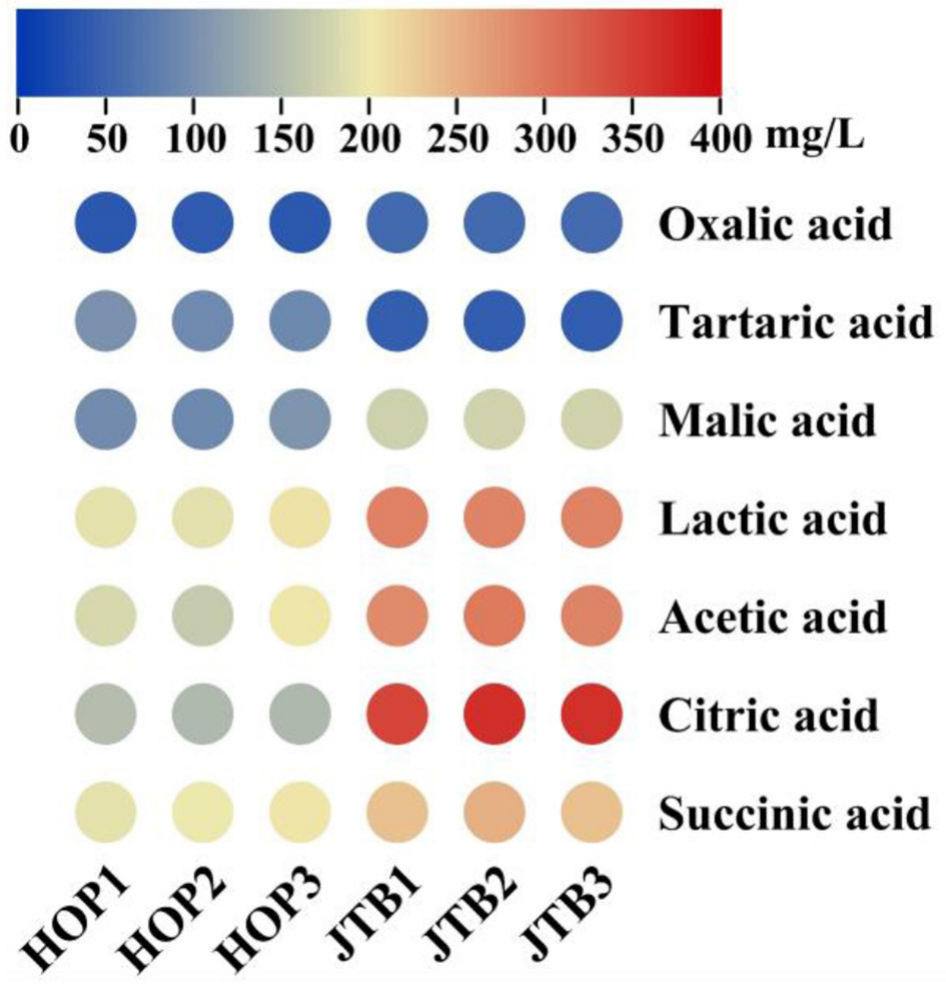
Heat map of seven organic acids.

Organic acids are important indicators of product quality and contribute to the organoleptic properties of beer, as well as being indicators of fermentation performance, so the organic acids (oxalic, tartaric, malic, lactic, acetic, citric, and succinic acid) in JTB and HOPB were determined (Figure 1). The total organic acid content of JTB (1445.38 mg/L), was 1.52-fold that of HOPB (*p* < 0.05). The main organic acids in both beers were lactic, acetic and citric acid and they differed significantly. The lactic, acetic and citric acid contents of JTB were 288.88 ± 1.02, 288.75 ± 7.34, and 356.44 ± 11.03 mg/L, respectively, 1.48-, 1.58- and 2.43-fold those of HOPB, respectively. The malic and succinic acid contents of JTB were 173.03 and 240.56 mg/L, 1.78- and 1.21-fold than those of HOPB, respectively. The tartaric acid content of JTB was significantly lower (0.44-fold) that of HOPB. The oxalic acid contents were similar, mainly because oxalic acid is primarily derived from the wort (38).

### 3.3. Analysis of volatile components

#### 3.3.1. Identification of volatile compounds

**Figure 2:**
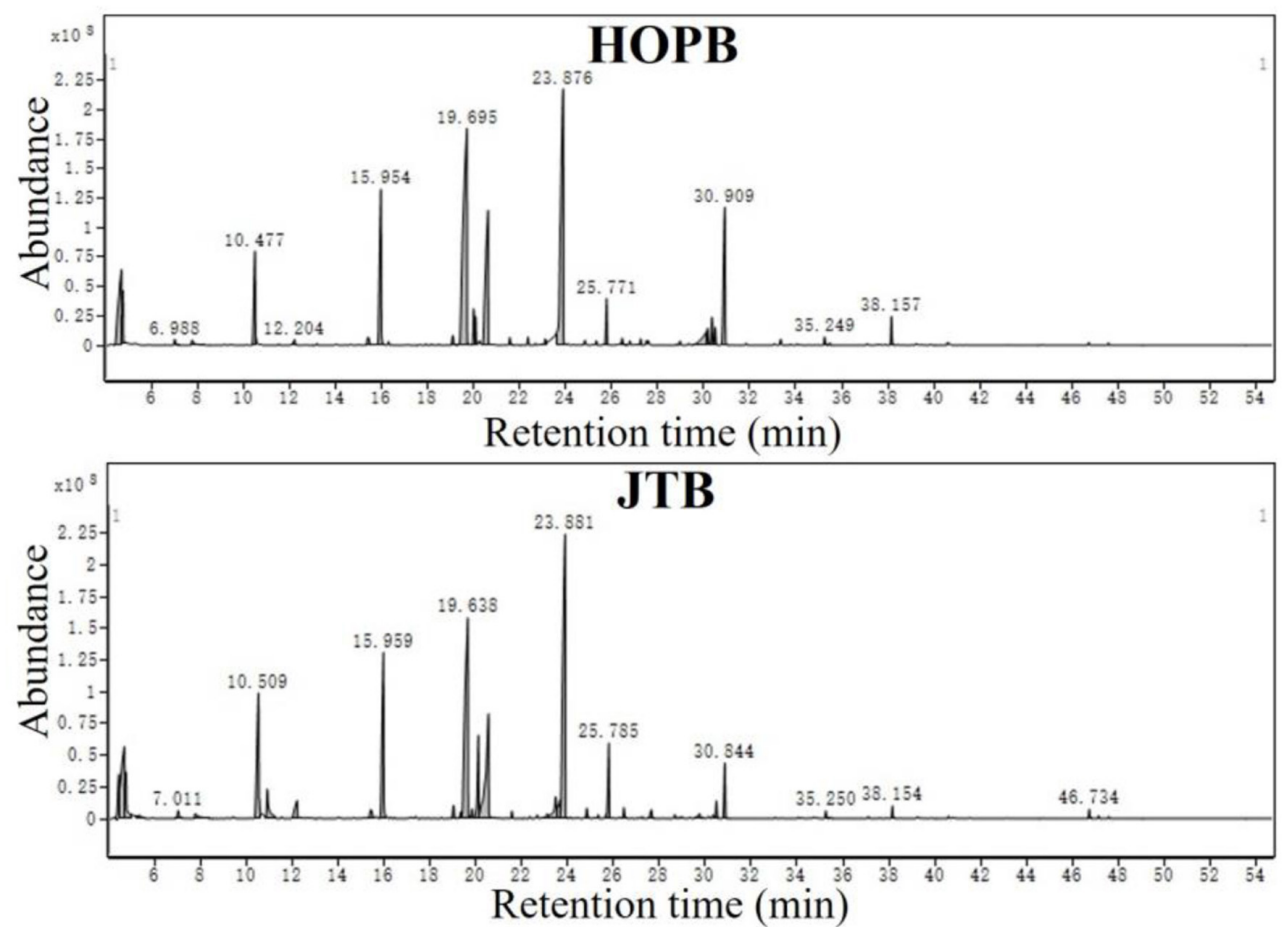
Total ion current (TIC) chromatograms of beer samples.

The volatile compounds in the beers were determined by HS-SPME-GC-MS; the GC-MS total ion current (TIC) chromatograms are shown in Figure 2. A total of 519 compounds was putatively identified by comparison with the NIST 14 database, of which 231 compounds had a NIST 14 Best Match score >70. The main volatiles found were 2-methyl-1-butanol, 3-methyl-1-butanol, 3-methyl butanol acetate, hexanoic acid ethyl ester, phenylethyl alcohol, octanoic acid ethyl ester, 2-phenylethyl acetic acid ester, decanoic acid ethyl ester, and dodecanoic acid ethyl ester. These substances are mainly produced by the fermenting yeast (39).

#### 3.3.2. Differential analysis of volatile compounds

**Figure 3:**
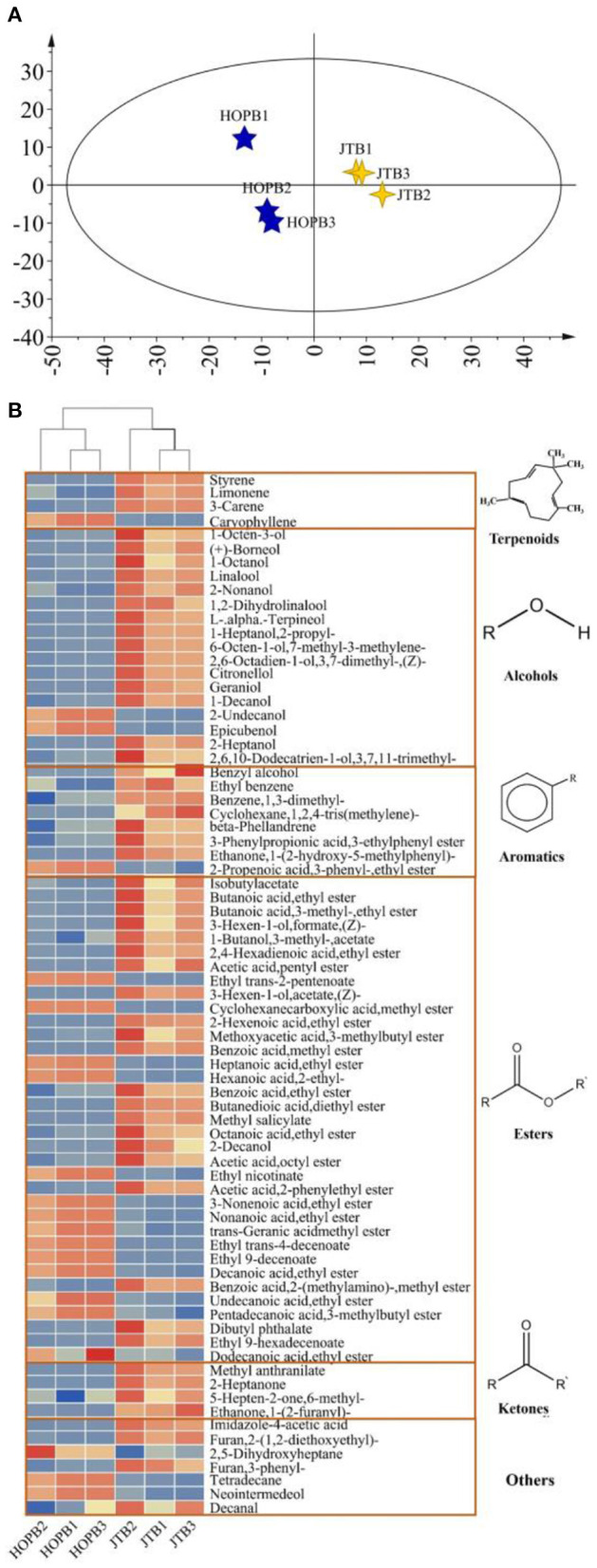
Differential volatiles analysis in beer samples. **(A)** Score plot from OPLS-DA model; **(B)** Heat map of the relative content of key difference volatile components.

OPLS-DA was used to compare the volatile profiles of the beers (Figure 3). The OPLS-DA score plot (Figure 3A) showed a clear difference between JTB and HOPB. To determine the most important differential volatile compounds between the beers, their VIP values were determined; with limits of VIP >1 and *p* < 0.05, 75 key volatile compounds were screened out. There were 17 alcohols, 36 esters, three ketones, four terpenes, eight aromatics and seven other compounds. Volatile esters were found to be the main differential components distinguishing the two beers. Esters are formed by a condensation reaction between alcohols and carboxylic acids, impart a fruity flavor to the beer and can strongly influence its overall flavor and style (40). A heat-map of the 75 key volatiles was plotted to visualize the differences between the beers (Figure 3B; red: content > mean, blue: content < mean) and a hierarchical cluster analysis (HCA) was performed to analyze clustering in the flavor profiles. As can be seen from the Figure 3B, the content of most volatiles in JTB is higher than that in HOPB. Cluster analysis enables a good classification of the same type of beer into one category.

#### 3.3.3. Analysis of major odor-active compounds

The relative odor activity value (ROAV) (41) was used to identify the contributions made by the 75 key volatiles to the overall flavor of the beers, finding 30 substances with ROAV ≥1 (Table 3), of which 22 were found in HOPB and 29 in JTB. The major contributors to the overall flavor of HOPB were linalool, ethyl 9-decenoate, 1-decanol, hexanoic acid ethyl ester and octanoic acid ethyl ester (ROAV 82.35, 76.23, 67.95, 32.19, and 21.12, respectively). The major contributors to the overall flavor of JTB were linalool, methyl anthranilate, 3-methyl-1-butyl acetate, 1-decanol, and hexanoic acid ethyl ester (ROAV 368.52, 73.85, 45.76, 44.77, and 43.61, respectively). Linalool, 1-decanol, and hexanoic acid ethyl ester were all relatively high in JTB and HOPB and contributed strongly to their aromas.

**Table 3:** Main odor-active compounds (ROVA≥1) in HOPB and JTB samples.

**ON**	**Compound**	**CAS**	**Threshold (μg/L)**	**HOPB (μg/L)**	**JTB (μg/L)**	**ROAV-HOPB**	**ROAV-JTB**
1	Linalool	78-70-6	6	494.07	2211.11	82.35	368.52
2	Methyl anthranilate	134-20-3	3	0.78	221.54	< 1	73.85
3	1-Butanol, 3-methyl-, acetate	123-92-2	210	3145.33	9608.58	14.98	45.76
4	1-Decanol	112-30-1	5	108.02	223.85	21.6	44.77
5	Hexanoic acid ethyl ester	51-79-6	230	7403.34	10029.19	32.19	43.61
6	Decanal	112-31-2	5	74.99	213.57	15	42.71
7	Acetic acid, 2-phenylethyl ester	103-45-7	210	3373.96	7265.75	16.07	34.6
8	Citronellol	68916-43-8	8	96.42	231.92	12.05	28.99
9	Octanoic acid, ethyl ester	106-32-1	900	19010.93	25919.57	21.12	28.8
10	2-Nonanol	821-55-6	3	50.08	64.26	16.69	21.42
11	Methyl salicylate	119-36-8	40	5.18	846.46	< 1	21.16
12	2-Nonanone	821-55-6	32	615.39	626.93	19.23	19.59
13	2-Heptanol	100-41-4	3	9.41	37.24	3.14	12.41
14	1-Octen-3-ol	542-30-3	6.12	31.64	60.58	5.17	9.9
15	Benzoic acid, ethyl ester	93-89-0	20	125.67	184.75	6.28	9.24
16	Benzoic acid, methyl ester	93-58-3	73	27.55	622	< 1	8.52
17	Acetic acid, octyl ester	112-14-1	800	3757	5170.33	4.7	6.46
18	Butanedioic acid, diethyl ester	27829-71-6	790	139.14	3318.44	< 1	4.2
19	Geraniol	106-24-1	40	27.34	132.24	< 1	3.31
20	Ethyl 9-decenoate	67233-91-4	100	7622.85	290.55	76.23	2.91
21	2,6-Octadien-1-ol, 3,7-dimethyl-, (Z)-	103-36-6	80	96.03	231.92	1.2	2.9
22	Butanoic acid, 2-methyl-, ethyl ester	7452-79-1	18	28.12	47.26	1.56	2.63
23	2-Heptanone	151301-57-4	140	11.21	363.76	< 1	2.6
24	Ethyl trans-4-decenoate	76649-16-6	112.29	7630.66	290.91	67.95	2.59
25	3-Hexen-1-ol, acetate, (Z)-	3681-71-8	31	8.5	74.85	< 1	2.41
26	Decanoic acid ethyl ester	110-38-3	1500	7622.91	2685.09	5.08	1.79
27	Butanoic acid ethyl ester	105-54-4	400	271.51	534.78	< 1	1.34
28	2-Ethylcaproic acid	149-57-5	230	1092.49	290.12	4.75	1.26
29	Dodecanoic acid, ethyl ester	106-33-2	400	798.82	451.17	2	1.13
30	Heptanoic acid, ethyl ester	106-30-9	400	1092.43	289.98	2.73	< 1

Methyl anthranilate and ethyl 9-decenoate were the components with the second highest ROAV values in JTB and HOPB, respectively, and both these compounds differed markedly between the two beers. This appears to be related to the different ingredients; methyl anthranilate is a key aroma component of jasmine tea (42, 43), and ethyl 9-decenoate is a key aroma component of hops (44). Linalool is the main contributor to the overall flavor of both beers (largest ROAV) and is a key flavor component of late hopped beers and teas; it provides a floral flavor to beer (45). Hexanoic acid ethyl ester and 1-decanol are volatile compounds produced during fermentation, which can provide floral and fruity aromas, respectively (20, 46).

### 3.4. Metabolomic analysis of non-volatile beer components by LC-MS

**Figure 4:**
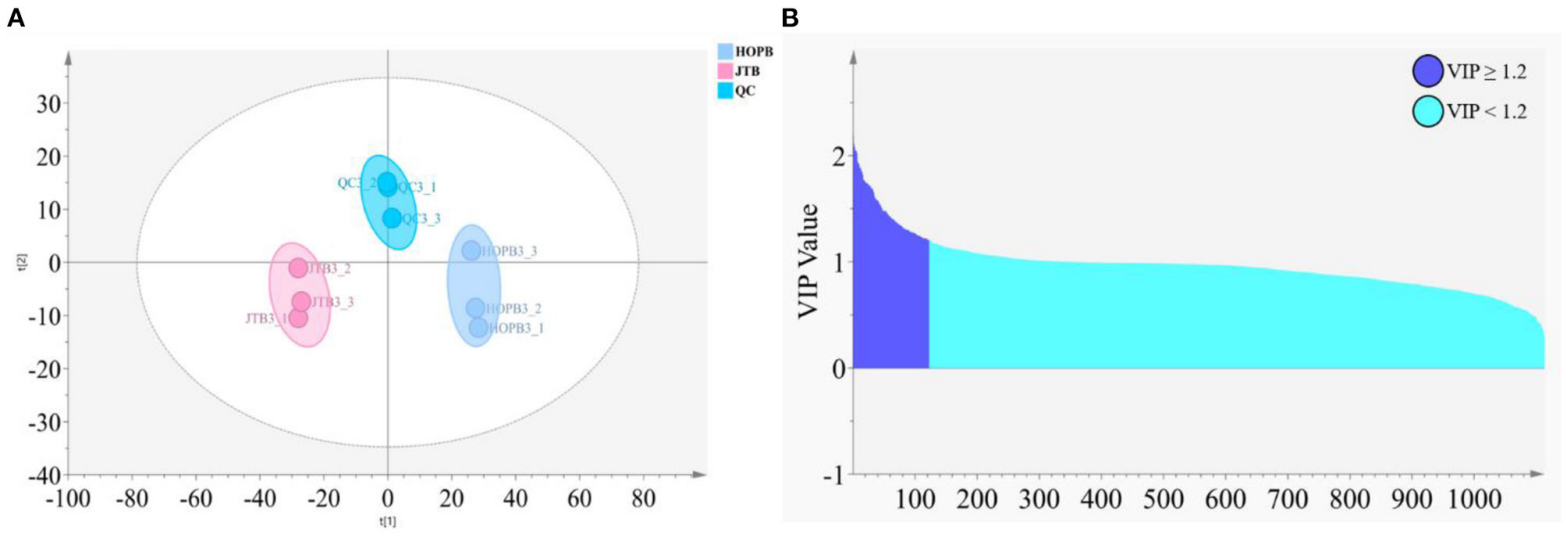
Analysis of differential metabolites in different beers. **(A)** PCA score plot; **(B)** PLS-DA S-plot of HOPB vs. JTB (purple: VIP ≥ 1.2, blue: VIP < 1.2).

After LC-MS data preprocessing, a total of 1,113 compound ion features were obtained from univariate and multivariate analysis. The QC samples were closely grouped in the PCA scores plot, indicating that the metabolomic analysis was reliable (Figure 4A). The JTB and HOPB samples were both closely grouped, but the two beer type groups were well separated, i.e., LC-MS analysis could clearly distinguish the two beer types. The criterion of PLS-DA VIP value ≥1.2 (Figure 4B) was used to screen for compounds that significantly differed between the two beers, and identified 123 differential compounds (Figure 4B, purple).

The 123 compounds were initially identified on the basis of their exact molecular masses and fragmentation spectra. Thirty-nine differential compounds were identified by comparing with the HMDB database (http://www.hmdb.ca/), laboratory standard libraries and previous reports (Table 4), namely, six bitter acids and derivatives, three amino acids, five phenolic acids, five organic acids, seven dimeric catechins, nine flavone/flavonol glycosides, and four others. Overall, JTE addition resulted in significant changes in metabolic profile.

**Table 4:** Forty tentatively identified metabolites between two groups of beer sample (in negative mode).

	**Compound**	**Rt (min)**	***m*/*z* [M–H]^−^**	**Fragments *m*/*z***	**Molecular formula**
1	Gluconic acid	0.87	195.05003	129.018, 75.008, 99.008	C_6_H_12_O_7_
2	Ribonic acid	0.88	165.0396	75.008, 129.018, 147.029	C_5_H_10_O_6_
3	Maltotetraose	0.9	665.2151	161.054, 179.056, 101.023	C_24_H_42_O_21_
4	Theanine	1.21	173.09214	85.028, 129.018	C_7_H_14_N_2_O_3_
5	Citramalic acid	1.56	147.02878	87.008, 85.028, 129.018	C_5_H_6_K_2_O_5_
6	Inosine	1.67	267.07369	135.03	C_10_H_12_N_4_O_5_
7	Glucogallin	1.85	331.06715	169.014, 211.025, 128.0340	C_13_H_16_O_10_
8	Gallic acid	2.03	169.01328	125.096, 126.100	C_7_H_6_O_5_
9	Xanthosine	2.03	283.06854	151.025	C_10_H_12_N_4_O_6_
10	(+)-Gallocatechin	3.7	305.06667	125.023, 167.035, 175.035	C_15_H_14_O_7_
11	Chlorogenic acid	4.38	353.08804	191.056, 192.059, 161.024	C_16_H_18_O_9_
12	5′-Methylthioadenosine	4.53	296.08232	134.046	C_11_H_15_N_5_O_3_S
13	EGC	4.932	305.06668	125.023, 179.034, 167.035	C_15_H_14_O_7_
14	2-Isopropylmalic acid	5	175.06039	115.039, 113.060, 85.065	C_7_H_12_O_5_
15	Procyanidin B1	5.03	577.13589	125.032, 289.072, 407.079, 161.024	C_30_H_26_O_12_
16	C	5.2	289.07184	245.082, 109.029.125.023	C_{1}{5}_H_{1}{4}_O_{6}_
17	Neochlorogenic acid	5.31	353.08799	191.056, 179.034, 135.044	C_16_H_18_O_9_
18	Procyanidin B2	5.41	577.13589	125.032, 289.072, 407.079, 161.024	C_30_H_26_O_12_
19	caffenic acid	5.61	179.03417	135.044, 179.034	C_9_H_8_O_4_
20	EC	5.77	289.07183	245.082, 109.029.203.071	C_15_H_14_O_6_
21	Epigallocatechin gallate	5.83	457.07799	169.013, 125.023, 305.067	C_22_H_18_O_11_
22	3-O-p-Coumaroylquinic acid	5.877	337.09307	173.045	C_16_H_18_O_8_
23	GCG	6.01	457.07799	169.013, 125.023, 305.067	C_22_H_18_O_11_
24	Apigenin 6-C-glucoside 8-C-arabinoside	6.03	563.14115	353.068, 383.078, 443.089, 473.110	C_26_H_28_O_14_
25	N-Acetyl-L-leucine	6.26	172.0971	130.088	C_8_H_15_NO_3_
26	4-Coumaric acid	6.47	163.03927	119.0049	C_9_H_8_O_3_
27	Rutin	6.47	609.14655	300.028, 301.033, 302.039	C_27_H_30_O_16_
28	Isoquercitrin	6.63	463.06504	169.013, 125.023, 300.028	C_21_H_20_O_12_
29	ECG	6.65	441.08292	169.013, 289.092.125.023	C_22_H_18_O_10_
30	N-Acetyl-DL-tryptophan	6.79	245.09317	230.082, 74.024, 116.034	C_13_H_14_N_2_O_3_
31	Kaempferol-3-O-D-galactoside	7.09	447.09369	284.033, 285.044, 488.097	C_21_H_20_O_11_
32	Kaempferol	8.7	285.04057	285.041	C_15_H_10_O_6_
33	Isoxanthohumol	8.95	353.1467	119.0493, 233.817, 59.0127	C_21_H_22_O_5_
34	Cohumulone	9.9	347.1863	235.134	C_20_H_28_O_5_
35	ad-humulone	10.131	361.20211	235.134, 36.137, 125.060	C_21_H_30_O_5_
36	iso-Cohumulone	10.19	347.18629	181.050, 251.129, 233.118	C_20_H_28_O_5_
37	iso-Cohumulone	10.394	347.18631	251.129, 181.050, 233.118	C_20_H_28_O_5_
38	iso-n/ad-humulone	10.466	361.20209	195.066, 265.145, 247.134	C_21_H_30_O_5_
39	Cohumulone	10.53	347.1863	278.116, 181.050, 251.129	C_20_H_28_O_5_
40	N-humulone	10.69	361.20214	292.133	C_21_H_30_O_5_

**Figure 5:**
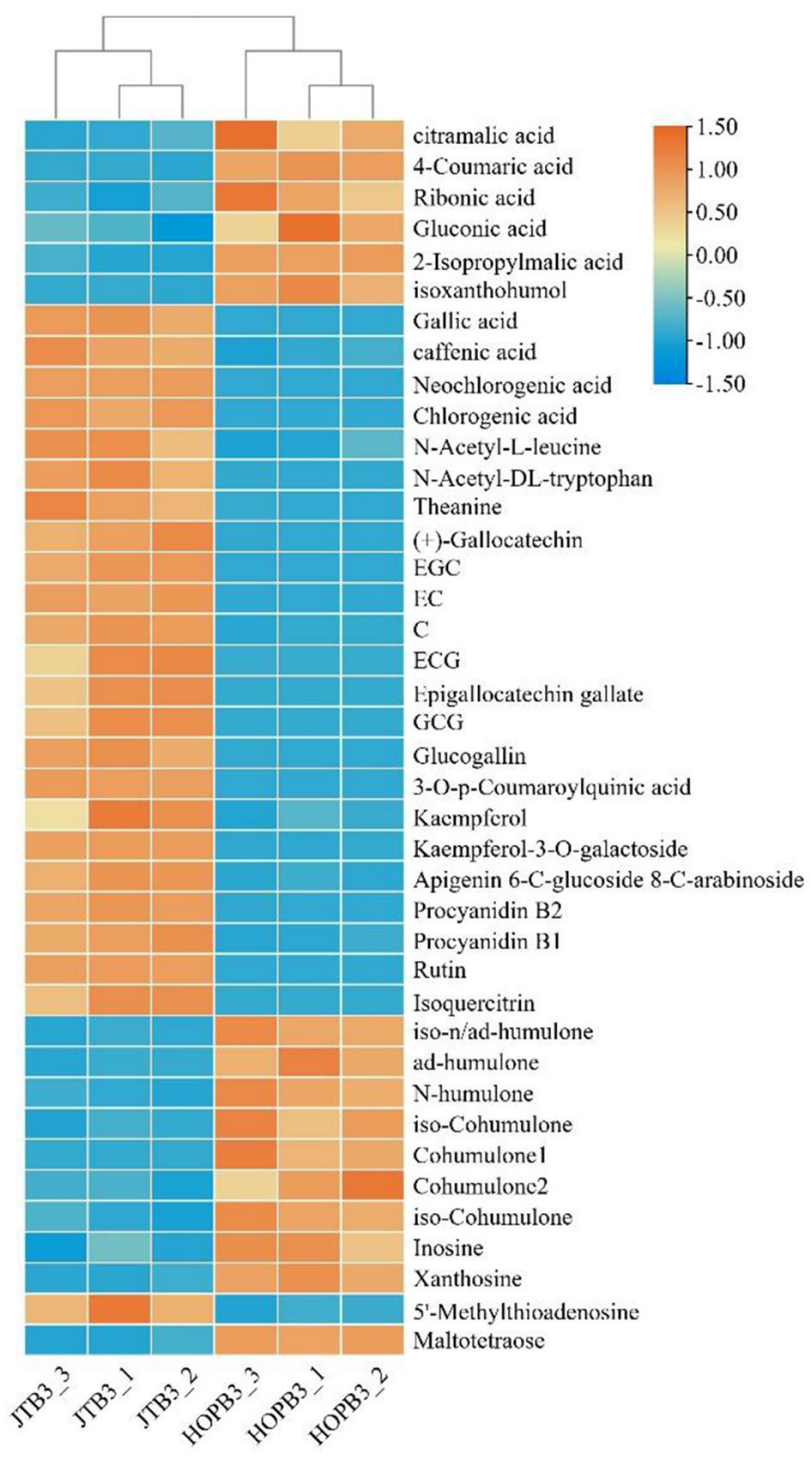
Heatmap analysis of critical metabolites in two beer samples. A color-coded scale grading from blue to orange corresponds to the content of critical metabolite shifting from low to high.

A heatmap was plotted to visualize the differential metabolites resulting from JTE addition (Figure 5). The content of phenolic acids, amino acids, dimeric catechins and flavone/flavonol glycosides in JTB was significantly higher than that in HOPB, whereas bitter acid and some organic acids were less abundant in JTB. These differences are consistent with the substitution of late hopping with the addition of JTE, as amino acids, dimeric catechins, flavone/flavonol glycosides and phenolic acids are abundant in tea, whereas bitter acids are only found in hops (47–50). Dimeric catechins, amino acids and flavone/flavonol glycosides from the JTE would give the beer a tea-like flavor, especially the theanine, which has an umami taste (51). The high content of iso-alpha-acids is the main reason for the bitterness of beer (52), which is consistent with the sensory evaluation results (see below). HOPB had a higher bitterness intensity.

### 3.5. Sensory evaluation

**Figure 6:**
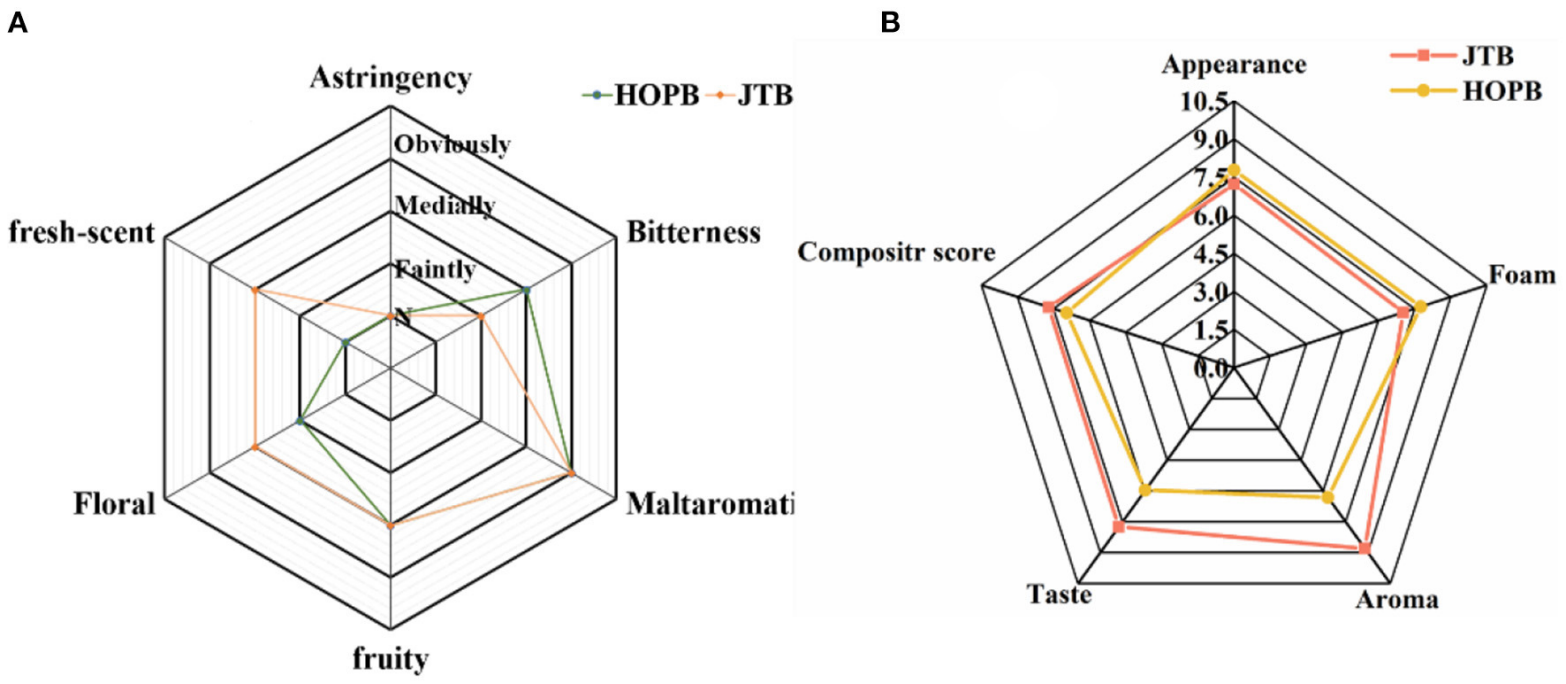
Results of the sensory profiling of the beer samples. **(A)** Sensory evaluation of beer flavor. **(B)** Rating evaluation of flavor intensity of beer characteristics.

Taste, flavor and other sensory attributes are the main determinants of beer quality (53). The appearance, foam, aroma, taste, and overall acceptability of the two beers were compared by sensory evaluation (Figure 6). There were differences in flavor intensity between the beers, with HOPB having a higher bitterness intensity and JTB having a higher floral and light fresh-scent intensity (Figure 6A); the aroma score for JTB was 8.8, 31.0% higher than that of HOPB. The aroma differences are consistent with the relative volatile profiles of the beers (section 3.2), i.e., JTB contained higher concentrations of linalool, α-terpineol and citronellol. The score for taste of JTB was 7.8, 32.5% higher than that of HOPB, whereas HOPB scored higher for bitterness. JTB had a richer, whiter and finer foam, with a score of 7.7, compared with 7.0 for HOPB. However, JTB scored lower for appearance than HOPB, because JTB was a little more turbid than HOPB. Overall, JTB had a higher organoleptic rating than HOPB. The taste of JTB was mellower, softer and with a pleasing tea flavor (Figure 6B). JTB had a higher aroma score than HOPB (*p* < 0.05), apparently because of the abundant floral and fresh fragrances released from JTE (54, 55). HOPB had a lower taste score and higher bitterness score than JTB, probably resulting from the late hopping of HOPB; late hopping enhances the bitterness of beer (56). The study of Oladokun et al. (57) showed a significant effect of polyphenol content on the perceived intensity and characteristics of bitterness, with higher polyphenol content resulting in stronger bitterness and poorer bitterness characteristics expression in beer. It is noteworthy that although the beer with tea extract added in this study had higher polyphenol content, the bitterness intensity of JTB did not become stronger.

### 3.6. Antioxidant capacity

**Figure 7:**
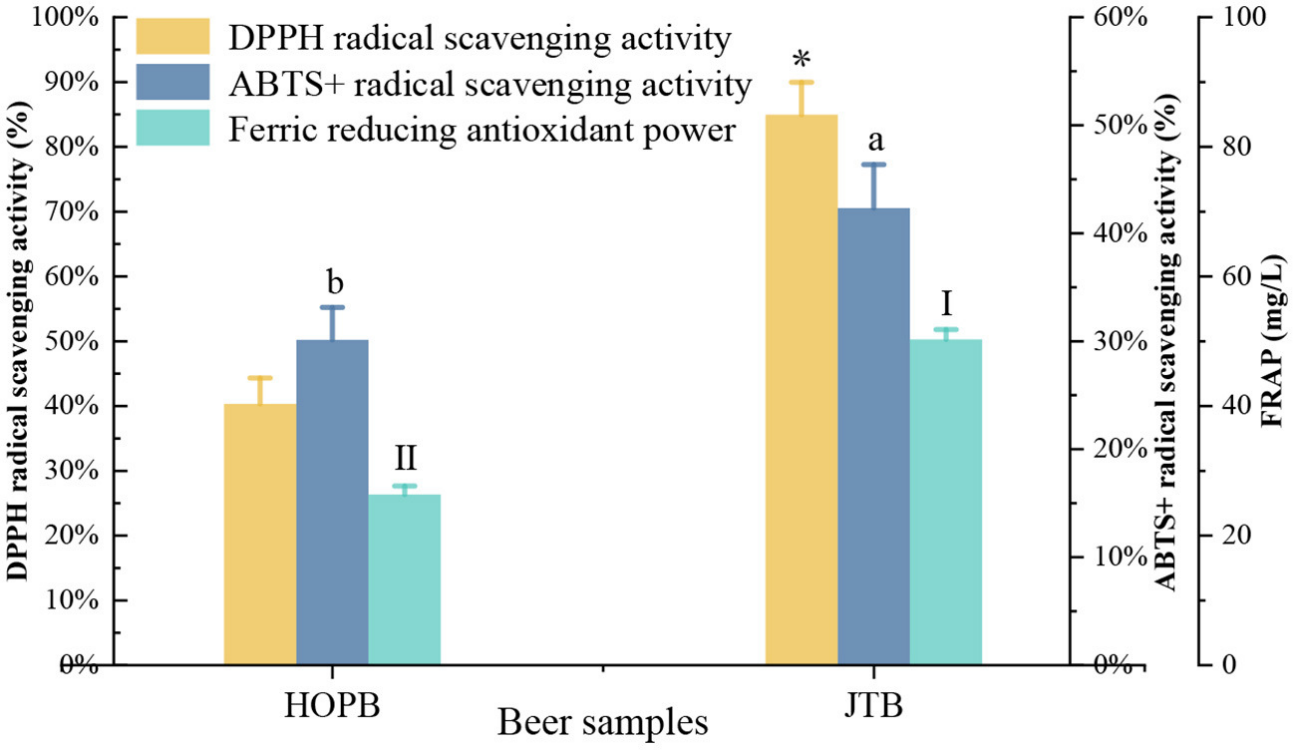
Antioxidant activity and polyphenol content. “a, b, I, II” and “^*^” Different letters in the same indexes indicate significant differences between mean values (*p* < 0.05). ABTS, 2,2′-azino-bis (3-ethylbenzothiazoline-6-sulphonic acid); DPPH, 2,2-diphenyl-1-picrylhydrazyl; FRAP, ferric reducing antioxidant power.

The antioxidant capacity influences the functional properties and the oxidation resistance of the beer during storage (30). The DPPH and ABTS+ radical scavenging capacities, and ferric reducing antioxidant power (FRAP) were determined to compare the antioxidant capacity of the beers (Figure 7). The DPPH, ABTS+, and FRAP capacities of JTB were 80.9, 42.3, and 50.3 mg/L, respectively, 52.6, 28.8, and 47.7% higher (*p* < 0.05) than those of HOPB, respectively.

The higher antioxidant capacities of JTB are consistent with its 1.6-fold higher phenolic content than HOPB; polyphenols, flavonoids, and flavonols account for the antioxidant capacity of beer (58, 59). Similarly, addition of fresh fruits during beer fermentation significantly enriched the content of phenolic compounds and increased the antioxidant capacity of the beer (60). Increased antioxidant capacity improves the storage stability of beer (36).

### 3.7. α-Amylase and α-glucosidase inhibition

**Figure 8:**
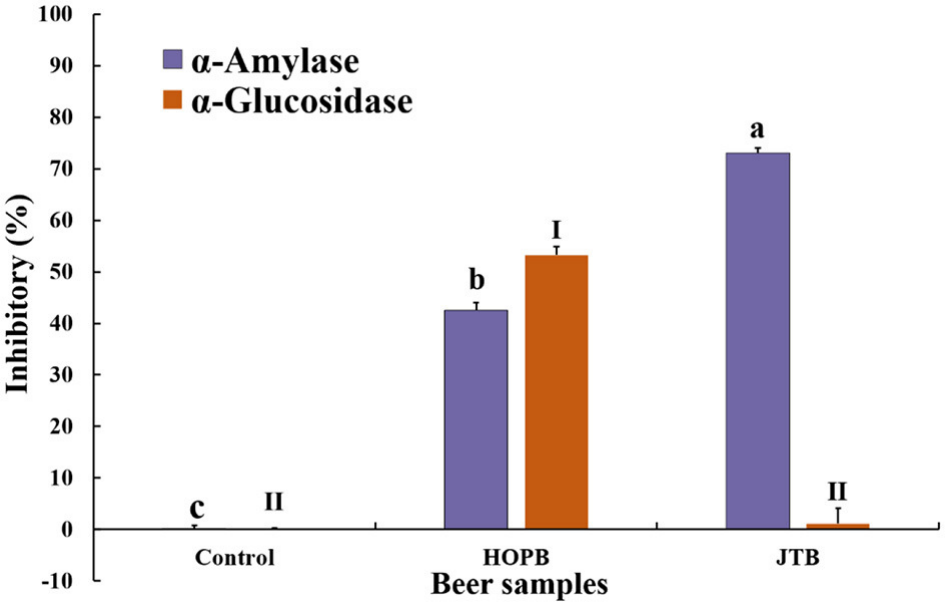
α-amylase and α-Glucosidase inhibitory effects by the two beers. “a, b, c, and I, II” Different letters in the same indexes indicate significant differences between mean values (*p* < 0.05).

α-Glucosidase and α-amylase are the key enzymes in the digestive system that hydrolyze dietary carbohydrates. Inhibition the two amylase can reduce and control postprandial blood glucose spikes, delaying hydrolysis of carbohydrates and suppressing postprandial hyperglycemia in prediabetes, diabetes, and obesity patients (61). The inhibitory effect on these digestive enzymes of the beers was determined (Figure 8); both beers inhibited α-amylase activity by 42.5 ± 1.5% (HOPB) and 72.1 ± 1.0% (JTB). α-Amylase inhibition by JTB was 30.5% higher than that of HOPB, probably because of its higher phenolic content; tea catechins strongly inhibit α-amylase activity (62, 63) and gallocatechin gallate is the strongest inhibitor among the catechins (61). HOPB inhibited α-glucosidase by 50.3%, whereas JTB had no significant effect. The characteristic compounds in hops inhibit both α-amylase (64) and α-glucosidase activity (65–67), which is consistent with the inhibition of both enzymes by HOPB.

## 4. Conclusion

In this study, beer was brewed with the addition of jasmine tea extract instead of hops at the late hopping stage. In general, the differences in physicochemical parameters between JTB and HOPB were not significant except for organic acid content and foam stability, but the overall sensory score of JTB was higher than that of HOPB and JTB had higher antioxidant capacity and polyphenol content.

The flavor volatiles in JTB and HOPB were distinctive; HOPB contained more abundant floral and fresh aroma compounds (e.g., nerol and methyl salicylate), whereas JTB contained more abundant green/grassy aroma compounds (e.g., hexanal). The differential compounds that distinguished the two beers were dimeric catechins, flavone/flavonol glycosides, and bitter acids and derivatives, which account for differences in sensory attributes and antioxidant capacity.

The overall sensory acceptability of JTB was higher than that of HOPB; JTB had a pleasant floral and fresh-scent aroma and softer taste, with a pleasant tea flavor and a taste. JTB had higher DPPH, ABTS and FRAP antioxidant capacities, which should result in better storage stability.

JTB has a stronger inhibitory effect on α-amylase activity than HOPB and is better able to regulate blood sugar levels. The consumption of this type of beer may therefore have a slowing effect on obesity.

Overall, adding jasmine tea extract instead of hops improved the overall sensory acceptability, foam stability and antioxidant capacity of the beer, as well as conferring a unique taste and flavor. This process modification has the potential to develop a novel application for tea in craft beer and compensate for the scarcity of aromatic hops in China.

## Data availability statement

The original contributions presented in the study are included in the article/supplementary material, further inquiries can be directed to the corresponding authors.

## Ethics statement

The studies involving human participants were reviewed and approved by Zhejiang Gongshang University Human Ethics Committee. The patients/participants provided their writen informed consent to participate in this study.

## Author contributions

Y-QX and CZ: conceived and designed the experiments. D-QC, CZ, Y-BH, and XZ: performed the experiments. D-QC, CZ, and Y-QX: analyzed the data. D-QC, CZ, Y-QX, PC, and J-FY: wrote and revised the paper. All authors have read and agreed to the published version of the manuscript.
